# Oligomannose-Coated Liposome as a Novel Adjuvant for the Induction of Cellular Immune Responses to Control Disease Status

**DOI:** 10.1155/2013/562924

**Published:** 2013-10-10

**Authors:** Naoya Kojima, Mariko Ishii, Yoko Kawauchi, Hideaki Takagi

**Affiliations:** Department of Applied Biochemistry, Tokai University, Hiratsuka, Kanagawa 259-1292, Japan

## Abstract

Professional phagocytic cells, such as dendritic cells, are mainly responsible for phagocytosis, antigen presentation, and cytokine secretion, which induce subsequent activation of T cell-mediated immunity. Thus, strategies that deliver antigens and stimulatory signals to the cells have significant implications for vaccine design. In this paper, we summarize the potential for liposomes coated with the neoglycolipids containing oligomannose residues (OMLs) as a novel adjuvant for induction of Th1 immune responses and CTLs specific for the encased antigen. OMLs preferentially take up peripheral phagocytic cells. In response to OML uptake, the cells secrete IL-12 selectively, enhance the expression of costimulatory molecules, and migrate into lymphoid tissues from peripheral tissues. OMLs also have the ability to deliver encapsulated protein antigens to the MHC class I and class II pathways to generate antigen-specific CTLs and Th1 cells, respectively, and lipid antigen to CD1d to activate NKT cells. Since administration of OML-based vaccines can eliminate an established tumor, inhibit elevation of the serum IgE level, and prevent progression of protozoan infections in several murine, human, and bovine models, OML-based vaccines have revealed their potential for clinical use in vaccination for a variety of diseases in which CTLs and/or Th1 cells act as effector cells.

## 1. Introduction

The initial recognition and phagocytosis of pathogens by professional phagocytic cells, such as dendritic cells (DCs) and macrophages, and subsequent antigen presentation are crucial in determining the type of effector T cells that mediate immune responses [1–3]. Thus, targeting and functional control of these phagocytic cells is a major objective in the design of antigen delivery systems for new vaccines [4–6]. Several criteria should be fulfilled by an antigen delivery system for a vaccine to induce optimal antidisease immune responses [6]. First, specific targeting of the phagocytic cells is required. Second, delivery of the antigen to MHC class I molecules is needed to induce CTLs, and the vector must also deliver stimulatory signals to antigen presenting cells (APCs), since antigen delivery to APCs without these signals can induce tolerance. Finally, the delivery system must also be able to accommodate the insertion of large antigenic sequences to enable broad MHC coverage.

So far, many cell surface molecules expressed on APCs have been examined to target and deliver antigens to APCs *in vivo*. One approach to facilitate the uptake and processing of exogenous soluble antigens has been to deliver antigens via immune complexes directed to receptors for IgG (Fc*γ*Rs) expressed on DCs, such as Fc*γ*RI, Fc*γ*RIIa, and Fc*γ*RIII [7–9]. Ligands for *α*M*β*2 integrin (CD11b/CD18) have also been tested for antigen delivery to APCs [10]. Alternatively, antigens can be targeted to members of the C-type lectin family expressed on DCs, such as DC-SIGN, DEC-205, LOX-1, dectin-1, and the mannose receptor (CD206) [11]. Using protein antigens conjugated to antibodies directed to these APC-specific molecules, *in vivo* targeting approaches have produced effective results. However, promising achievement depends on overcoming the weak immunogenicity of many antigens.

On the other hand, development of particulate adjuvants and vaccines has increased during the past 30 years [4]. This has been invoked by the fact that all pathogens are particulate and that microparticles such as liposomes and polymer particles are efficiently uptaken by professional phagocytic cells. In addition, some of the particulate formulations tested were shown to have the potential for inducing helper and cytotoxic T cell responses [12]. Liposomes are vesicles formed from phospholipid bilayers and can encapsulate hydrophilic large antigenic sequences and immunomodulatory factors into the internal space of the vesicles, while amphiphilic antigens can be inserted into the bilayer and thus can serve as potent delivery vehicles [13–15]. Many types of liposomes have been tested in attempts to increase immune responses [15–17]. Enhanced uptake of liposomes by phagocytic cells indicates an adjuvant effect in inducing cellular immune responses, including the generation of antigen-specific CTLs [18, 19]. Cationic liposomes provide an important example of cellular delivery, since the positive charge on the liposome surface enhances uptake and subsequent CTL generation. Preferential delivery of antigen-containing liposomes to phagocytic cells can be facilitated by agents that bind selectively to molecular structures on the surface of the targeted cells. Conjugation of liposomes with antibodies and recombinant proteins, such as soluble forms of cell surface receptors or their ligands, can be used to target liposomes to receptors on cells [15, 20, 21]. Since C-type lectins expressed on APCs recognize certain structures of carbohydrates, such carbohydrates are also candidates for conjugation of liposomes to target APCs. 

We have developed a new liposome-based antigen delivery system for control of diseases in which CTLs and/or Th1 cells act as effector cells, using oligomannose-coated liposomes (OMLs) as a novel adjuvant and antigen delivery agent [22–25]. Feasibility studies of OML-based vaccines have revealed their potential for clinical use in vaccination for diseases in which CTLs and/or Th1 cells act as effector cells. This paper provides an overview of the current progress of the OML-based vaccine.

## 2. Carbohydrate-Coated Liposomes Are Suitable for Delivery of Antigens to Phagocytic Cells

The recognition and phagocytosis of pathogens and their subsequent destruction are an important mechanism of immune defense. Pathogens share similar structures that are known as pathogen-associated molecular patterns (PAMPs). Pattern recognition receptors (PRRs), such as Toll-like receptors (TLRs) and C-type lectin receptors (CLRs), are involved in the recognition of pathogens for the induction of immune responses [26–28]. Among them, CLRs are particularly important for the recognition and uptake of glycosylated antigens, including pathogens, into cellular compartments of APCs, such as DCs, leading to processing and presentation of antigens on MHC class I and class II molecules [29, 30]. Therefore, CLRs on phagocytic cells are often called endocytic/phagocytic receptors or antigen-uptake receptors. In addition, some CLRs directly or indirectly trigger distinct signaling pathways that induce expression of specific cytokines, which then determine T cell polarization [31]. The abilities of CLRs to mediate endocytosis and intracellular sorting of ligands and to trigger intracellular signaling implicate that CLRs are potentially useful for antigen targeting. Antibody-mediated targeting to APC-restricted CLRs, such as CD206, DEC-205, DC-SIGN, dectin-1, dectin-2, and DNGR-1, can be used to deliver antigens to late endosomal-lysosomal compartments of APCs, followed by the generation of MHC complexes of antigenic peptides to induce antigen-specific CD4^+^ and/or CD8^+^ T cell responses [32–34]. However, targeting of CLRs with these antibodies cannot deliver stimulatory signals to APCs, and therefore, an additional adjuvant is required for the maturation and activation of APCs [34]. Since CLRs recognize distinct carbohydrates expressed on pathogens, the use of carbohydrates that are preferentially recognized by CLRs as targeting signals may provide a more sophisticated alternative, and carbohydrate-coated liposomes with encapsulated antigens may be a more suitable antigen delivery vehicle to target CLRs than antibodies.

## 3. Oligomannose-Coated Liposomes Are Preferentially Ingested by Phagocytic Cells *In Vivo*

Carbohydrate structures containing terminal mannose are aberrant components of the cell surface of living mammalian cells. On the other hand, such structures are abundant and highly conserved on the surface of many pathogens. The professional phagocytic cells express a number of CLRs with an EPN motif in their carbohydrate recognition domain [30]. These CLRs facilitate the binding and uptake of ligands with terminal mannose, fucose, and *N*-acetylglucosamine residues and are thought to participate in the capture of pathogens [25, 35]. This activity was first observed for the CD206 but has now been focused on the family of DC-SIGN (CD209), Langerin (CD207), and dectin-2, since expression of these CLRs is restricted to professional phagocytic cells, such as immature DCs and macrophages [36–39]. These CLRs, which are restricted to phagocytic cells, are thought to be targets for specific antigen delivery. It has been shown that antigens modified by mannose residues can be preferentially delivered into APCs via CD206- or CD209-mediated uptake of antigens, resulting in effective antigen presentation to T cells [40]. These results led us to predict that antigen-carrying liposomes coated with mannose residues would induce strong immune responses *in vivo*. There are several examples in the literature of the use of liposomes decorated with mannose or mannan to target the mannose receptors on DCs [41–43], but the majority of studies have only been performed *in vitro*. 

**Figure 1:**
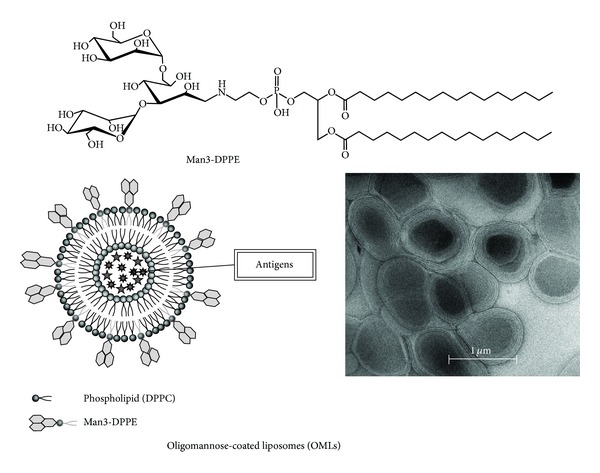
Representation of an OML. Man3-DPPE was prepared by reductive amination of an aldehyde group at the end of the mannotriose (Man3) with an amino group of DPPE. OMLs are prepared from DPPC, cholesterol, and Man3-DPPE at a molar ratio of 10 : 10 : 1 by intense vortex dispersion with antigen-containing PBS and are extruded through a 1 *μ*m pore membrane.

For *in vivo* targeting of APCs, we used OMLs comprised of dipalmitoylphosphatidylcholine (DPPC), cholesterol, and a neoglycolipid containing oligomannose residue at a 10 : 10 : 1 molar ratio. The oligomannose-containing neoglycolipid was prepared by conjugation of mannotriose (Man3) or mannopentaose (Man5) with dipalmitoylphosphatidylethanolamine (DPPE) by reductive amination [24]. The purities of the neoglycolipids were at least 95%. Neoglycolipid-coated liposomes consisting of cholesterol, DPPC, and a neoglycolipid were prepared as follows. A chloroform-methanol (1 : 1, v/v) solution containing 1.5 *μ*mol of DPPC, 1.5 *μ*mol of cholesterol, and 0.15 *μ*mol of neoglycolipid was added to a flask and evaporated to prepare a lipid film containing neoglycolipid. PBS (150 *μ*L) containing antigens was added to the dried lipid film and multilamellar vesicles were prepared by intense vortex dispersion. The vesicles were extruded 10 times through a 1 *μ*m pore polycarbonate membrane [24]. The mean (±SD) molar ratio of DPPC, cholesterol, and neoglycolipid in the liposomes was 1.00 : 1.09 ± 0.21 : 0.11 ± 0.03, and the particle sizes of the liposomes ranged from about 850 to 1450 nm, with a mean of 1265 nm. As shown in Figure 1, the hydrophobic moiety of the neoglycolipid permits easy incorporation into the lipid bilayer of multilamellar vesicles (liposomes) comprised of DPPC and cholesterol. On the other hand, the carbohydrate moieties of neoglycolipids on OMLs are exposed on the aqueous face due to their hydrophilicity, since OMLs aggregate with concanavalin A, a mannose-binding lectin. This also indicates that OMLs could be used in targeting mannose-recognizing molecules, such as CD206 and CD209, which are expressed on APCs *in vivo*. It should be noted that the structures of Man3 and Man5 are part of high mannose-type N-linked oligosaccharides, which are ubiquitously present in eukaryotic cells, and the other components of OMLs are also ubiquitously distributed inert materials. Therefore, OMLs do not exhibit antigenicity, probably due to their recognition as “self” or “harmless foreign” particles.

**Figure 2:**
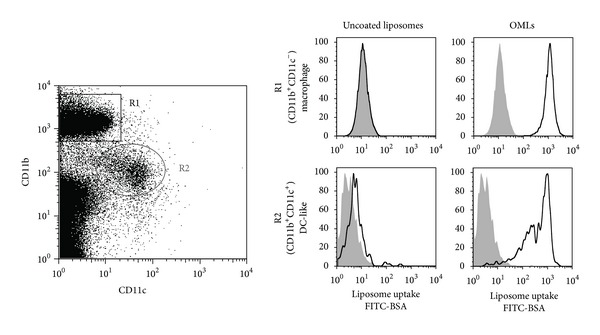
Uptake of OMLs by peritoneal phagocytic cells. Peritoneal phagocytic cells are classified into CD11b^+^CD11c^−^ (R1) and CD11b^+^CD11c^+^ (R2) cells, which belong to macrophage and dendritic cell lineages based on the cell surface markers, respectively. Both macrophage and DC-like cells effectively took up OMLs, when OMLs were administrated into peritoneal cavity.

Initial evidence supporting the hypothesis that OMLs could be used as delivery vehicles targeted to phagocytic cells *in vivo* was based on preferential uptake of FITC-BSA encasing OMLs (FITC-OMLs) by peritoneal phagocytic cells. Most of CD11b^+^ peritoneal phagocytic cells took up FITC-OMLs within 1 h after injection of the OMLs into the peritoneal cavity of mice (Figure 2), and the FITC-OML-containing cells subsequently appeared in extranodal lymphoid tissues within 24 h [44]. In addition, the ingested FITC-OMLs also appeared in late endosomes and lysosomes. On the other hand, FITC-BSA encased by liposomes without a neoglycolipid coating (FITC-BLs) was barely ingested by the peritoneal phagocytic cells. Comparison of the uptake efficiencies of FITC-OMLs and FITC-BLs, based on intensities of fluorescent signals, showed that the peritoneal phagocytic cells took up FITC-OMLs at least 15-fold more effectively than FITC-BLs. FITC-OMLs but not FITC-BLs also accumulated into the draining lymph nodes following subcutaneous administration. These results strongly indicated that OMLs could act as a suitable vehicle for *in vivo* targeting of APCs.

SIGNR1 (CD209b), a mouse homologue of human DC-SIGN, participates in intraperitoneal uptake of OMLs by peritoneal phagocytic cells [45]. In addition, complement receptor 3 (CR3; CD11b/CD18) also acts as the physiological receptor of OMLs [46]. Recently, it has been reported that C3 deposition on microorganisms is initiated by the interactions of SIGNR1 with polysaccharides on the microorganism and the complement C1 subcomponent, followed by activation of the classical complement pathway [47]. Therefore, SIGNR1 may promote uptake of OMLs through the activated complement pathway, and this step may be essential in the development of robust cell-mediated immune responses against the antigens in OMLs [35].

## 4. Phagocytic Cells Are Stimulated in Response to *In Vivo* Uptake of OMLs

To achieve effective antigen delivery to the APCs for vaccination, the vector must have the capacity to deliver stimulatory signals to APCs [6]. To determine whether OMLs could deliver stimulatory signals to APCs, we evaluated the adjuvant activity of OMLs based on the expression of costimulatory molecules and MHC class II molecules on peritoneal phagocytic cells. Following *in vivo* uptake of OMLs, expression of CD40, CD80, CD86, and MHC class II molecules on the OML-containing cells was clearly enhanced without any additional adjuvants or encased antigens [48]. We also demonstrated that expression of CCR7 was clearly upregulated on the cells with ingested OMLs [49]. Expression of CCR7 is essential for trafficking of APCs to secondary lymphoid organs and presentation of antigens to naïve T cells [50]. Indeed, we found that peritoneal phagocytic cells migrated to the spleen from the peritoneal cavity within 18 h after OML uptake. In addition, a fraction of peritoneal phagocytic cells can differentiate into mature APCs with a DC-like phenotype [51]. Therefore, OML uptake can induce activation, differentiation, and maturation of APCs, and thus OML itself exhibits potency as an adjuvant. 

**Figure 3:**
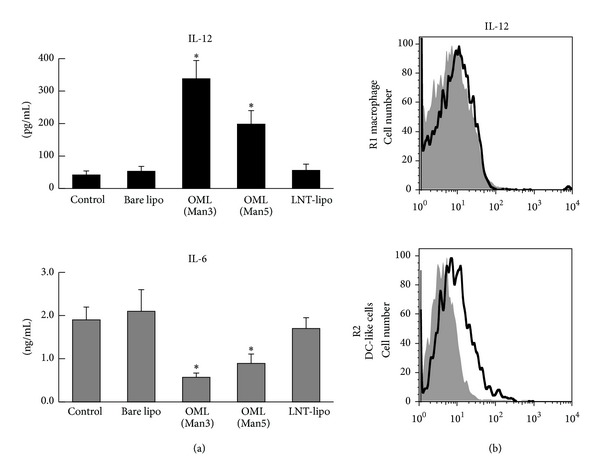
Preferential secretion of IL-12 from phagocytic cells. (a) The peritoneal phagocytic cells were collected 1 h after the administration of liposomes into peritoneal cavity and cultured for 18 h. IL-12 and IL-6 secreted into culture media were determined. Note that specific IL-12 production and suppression of IL-6 production were observed in the cells that took up OMLs (Man3 or Man5), while carbohydrate uncoated liposomes (Bare lipo) and liposomes coated with lacto-N-tetraose, which has terminal galactose residue (LNT), were not affected in secretion of IL-12 and IL-6. (b) Intracellular staining of IL-12 of peritoneal phagocytic cells that ingested OMLs. IL-12 was produced by CD11b^+^CD11c^+^ (R2 in Figure 2) cells.

We have also demonstrated that peritoneal phagocytic cells preferentially produce IL-12 in response to OML uptake (Figure 3) [45]. Further analyses have revealed that peritoneal phagocytic cells with a DC-like phenotype, which express CD11c, CD86, and MHC class II, are responsible for the production of IL-12 [49]. These findings provide important evidence supporting the activation of APCs by OMLs. Since IL-12 is critical for the development of Th1 cells and the initiation of cell-mediated immune responses [50], the production and secretion of IL-12 from OML-containing DC-like cells may be crucial in the induction of OML-stimulated Th1 immune responses. Interestingly, the production of IL-12 from peritoneal cells was clearly augmented, while the production of IL-1*β* and IL-6 from these cells was suppressed following incorporation of OML (Figure 3). It is generally accepted that TLRs play a key role in pathogen recognition and activation of APCs. The binding of PAMPs to TLRs triggers signal transduction events that lead to the activation of mitogen-activated protein kinases and transcription factors, such as NF-*κ*B, resulting in proinflammatory responses, including the production of proinflammatory cytokines, such as TNF-*α*, IL-12, IL-6, and IL-1*β* [27]. Indeed, TLR ligands, such as CpG-ODN or LPS, induce the production of IL-12 and high levels of IL-1*β* and IL-6 from peritoneal phagocytic cells [45]. In addition, peritoneal phagocytic cells from TLR4-dysfunctional C3H/HeJ mice also produced IL-12 upon uptake of OMLs. Therefore, OMLs might activate APCs through particular signaling pathways that are distinct from those triggered by TLRs, leading to specific production of IL-12 and to suppression of IL-6 and IL-1*β* production. Although the detailed signaling pathways triggered by OMLs remain to be elucidated, novel adjuvant activities of OMLs might enhance immunogenicity of antigens and promote DC maturation, leading to encased-antigen-specific immune responses.

## 5. OMLs Deliver the Encased Antigen to MHCs to Induce Antigen-Specific Th1 Immune Responses and the Production of Cytotoxic T Cells

In order to reject invading pathogens and cancer cells, the concomitant activation of both CD8^+^ and CD4^+^ T cells and the selective activation of CD4^+^ T cells with helper function are required [52]. In general, exogenous antigens presented by MHC class II molecules are intended for CD4^+^ T cells, while internal antigens from components of virus-infected cells and cancer antigens are presented on MHC class I molecules for the activation of CD8^+^ T cells. Therefore, the vehicles must deliver the administered exogenous antigens to both MHC class I and class II pathways. We demonstrated the usefulness of OMLs as carriers for the delivery of encased antigens to both the MHC class I and class II pathways. This ability was evaluated using ovalbumin- (OVA-) specific T cell receptor transgenic OT-I (specific for H-2Kb/OVA_257–264_) and OT-II (specific for H2Ab/OVA_323–339_) mice, respectively. The peritoneal phagocytic cells that were treated with OMLs containing entrapped OVA (OML/OVA) led to significant increases in IFN-*γ* production from CD8^+^ T cells from OT-I mice or CD4^+^ T cells from OT-II mice compared with cells that were treated with OVA alone, strongly indicating that the OML/OVA-ingesting phagocytic cells effectively activated OVA-specific CD8^+^ and CD4^+^ T cells via presentation of OVA peptides on MHC class I and class II molecules, respectively [53]. 

We also demonstrated that OMLs could generate encased-antigen-specific CTLs [24]. C57BL/6 mice were immunized biweekly three times with OML containing 1 *μ*g OVA, and then spleen cells were isolated from the mice one week after the last immunization. CD8^+^ cells were prepared from spleen cells after *in vitro* stimulation of spleen cells with OVA, cocultured with target cells (E.G7-OVA or EL4), and the cytotoxicity was measured. Only CD8^+^ cells from mice immunized with OML/OVA, and not those from mice treated with OVA-containing naked liposomes, exhibited strong cytotoxicity against E.G7-OVA (OVA-transfected EL4), but not against parental EL4 tumor cells. In addition, only mice immunized with OML/OVA rejected E.G7-OVA, but not parenteral EL4. On the other hand, when the EL4 tumor cell lysate was used for tumor antigens, all mice that received EL4 tumor cell lysate-containing OML completely rejected the EL4 tumor, whereas EL4 tumor growth was seen in the mice which received EL4 tumor cell lysate-containing naked liposomes. Identification of tumor-associated antigens (TAAs) that elicit tumor-specific CTL responses facilitates the development of cancer immunotherapy. Several groups have attempted to develop a vaccine strategy using tumor cell lysates as a possible TAA source, since TAAs have yet to be identified for most human cancers. Therefore, our OML-based approach may be promising in TAA-based cancer immunotherapy.

In contrast, the induction of antigen-specific Th1 immune responses was demonstrated using an infection model of mice with *Leishmania major* [22, 54]. The Th1 immune response is the key event in preventing *L. major* infection, and in a resistant mouse strain the infection predominantly induces the onset of a Th1 immune response that leads to recovery from infection. Susceptible mouse strains, such as BALB/c, preferentially develop a Th2 immune response characterized by enhanced expression of IL-4, and exhibit nonhealing lesions and disease progression [55, 56]. Intraperitoneal immunization of BALB/c mice with OMLs with encased soluble leishmanial antigen enhanced antigen-specific IFN-*γ* production, suppressed IL-4 production from spleen cells, and protected against subsequent *L. major* infection, indicating that Th1 immune responses predominated over Th2 immune responses in the OML-treated BALB/c mice. Administration of antigen alone or antigen-encased naked liposomes failed to induce similar immune responses. Collectively, these results indicate that OMLs can be used as an effective antigen delivery vehicle and as an APC activation system for immunotherapy with activation of both Th1 cells and CTLs.

## 6. OMLs Deliver the Lipid Antigen to CD1d to Activate NKT Cells

A subpopulation of T cells, referred to as invariant natural killer T (iNKT) cells, can recognize glycolipid antigens presented by the MHC class l-like molecule, CD1d [57, 58]. The glycolipid antigen, alpha-galactosylceramide (*α*GC), has been used as an exogenous ligand for CD1d to stimulate mouse V*α*14 NKT and human V*α*24 NKT cells [59]. The antitumor and anti-infectious properties of *α*GC have also attracted attention, since *α*GC-reactive iNKT cells rapidly produce significant amounts of Th1 and Th2 cytokines in response to stimulation, which can subsequently activate other immune cells, such as natural killer (NK) cells. By analogy, with the effective delivery of liposome-encased protein antigens to MHC class I and class II molecules, we hypothesized that OMLs could also be used as a vehicle for preferential delivery of lipid antigens, such as *α*GC, to CD1d to activate iNKT cells. 

To this end, we prepared *α*GC-containing liposomes coated with Man3-DPPE (*α*GC-OMLs) and with no coating (*α*GC-BLs), which consisted of cholesterol, DPPC, Man3-DPPE, and *α*GC at molar ratios of 10 : 10 : 1 : 1 and 10 : 10 : 0 : 1, respectively, and compared the *in vitro* and *in vivo* responses of iNKT cells to *α*GC-OMLs and to *α*GC-BLs. GC-OMLs stimulated iNKT cells to produce IFN-*γ* more efficiently than *α*GC-BLs or soluble *α*GC *in vitro* [60]. This property of *α*GC-OMLs appeared to be due to preferential uptake by phagocytic cells, relative to *α*GC-BLs or soluble *α*GC. Indeed, we showed that *α*GC-OMLs were preferentially incorporated into splenic DCs as well as BMDCs* in vitro*, compared with *α*GC-BLs. Systemic administration of *α*GC-OMLs led to more rapid and continuous IFN-*γ* release in sera, compared to *α*GC-BLs or soluble *α*GC, and also resulted in a more dramatic expansion of iNKT cells in peripheral blood, compared with *α*GC-BLs or soluble *α*GC [60]. Since *in vivo* activation of iNKT cells with *α*GC stimulation is known to be characterized by rapid expansion [61], *α*GC-OMLs activate iNKT cells *in vivo* with much higher efficacy than *α*GC-BLs or soluble *α*GC via effective delivery of *α*GC to CD1d. Collectively, our current results indicate that *α*GC-formulated particles modified by carbohydrate ligands of DC-restricted CLRs can be used as more appropriate *in vivo* and *ex vivo* delivery systems of lipid antigens to APCs to activate iNKT cells, compared with particles lacking carbohydrates.

## 7. Therapeutic Application of OML-Based Vaccines

The increasing knowledge of the effects of OMLs on APCs led us to test an OML-based vaccine for control of diseases in which antigen-specific Th1 cells and/or CTLs are the main effectors. The therapeutic efficacies of the OML-based vaccines tested to date are summarized in Table 1. One promising finding was initially obtained in a murine model of *L. major *infection, as described above [22, 54]. Preliminary studies of the effects of OML-based vaccines administered via the subcutaneous route have also been performed using soluble protozoan lysates of *Toxoplasma gondii*, *Trypanosoma brucei gambiense*, and *Babesia rodhaini* in the corresponding protozoan infections in mice, and these studies indicated that the OML-based vaccine is effective against protozoan infection in mice. We also showed that OMLs could also control the infection of *Neospora caninum *in mice using recombinant antigens. Subcutaneous administration of OMLs with encased recombinant *Neospora *antigen NcGRA7 was able to prevent transition of infection to the brain and transplacental vertical transmission [23]. In addition, immunization with OMLs and encased apical membrane antigen 1 of *Neospora* reduced offspring mortality [62]. Based on these protective effects of OML-based vaccines against protozoan infections in murine models, we assessed the effects of OML-based vaccine in farm animals. Subcutaneous administration of OMLs with encased recombinant NcGRA7 can induce protective immune responses to *N. caninum* in cattle [63], indicating that OML-based vaccines may be useful for prophylaxis against some infectious diseases in animal husbandry.

Type I allergic reactions depend on allergen-specific Th2 cells, which produce IL-4 and IL-5 and promote synthesis of IgE [64]. IFN-*γ* produced by Th1 cells inhibits the development and activation of Th2 cells and prevents IgE production [65, 66] and OMLs can induce encased-antigen-specific Th1 immune responses. Consequently, OML-based vaccines with encased allergens are expected to exhibit antiallergic effects. One promising antiallergic effect of an OML-based vaccine has been demonstrated in an animal model for Japanese cedar pollinosis [25]. Immunization of OMLs with entrapped Cry j 1, which has been identified as a major allergen in Japanese cedar pollen, has been shown to inhibit the elevation of the serum IgE level elicited by Cry j 1 administration in both nonsensitized mice and Cry j 1-presensitized mice. This inhibitory effect might occur through a shift from a Th2 immune response to an allergen-specific Th1 immune response, since Cry j 1-specific IgG1, which is mediated by Th2 cells, was significantly reduced, whereas Cry j 1-specific IgG2a, which is produced by Th1 cells, was increased in sera from OML-based vaccinated mice. Recently, it was demonstrated that intranasal administration of OML-based vaccines could induce both mucosal and systemic immune responses and suppress the development of allergic diarrhea induced by oral OVA administration [67, 68]. Therefore, OML-encased allergens may serve as immunotherapeutic agents to control allergic diseases including food allergies. 

**Figure 4:**
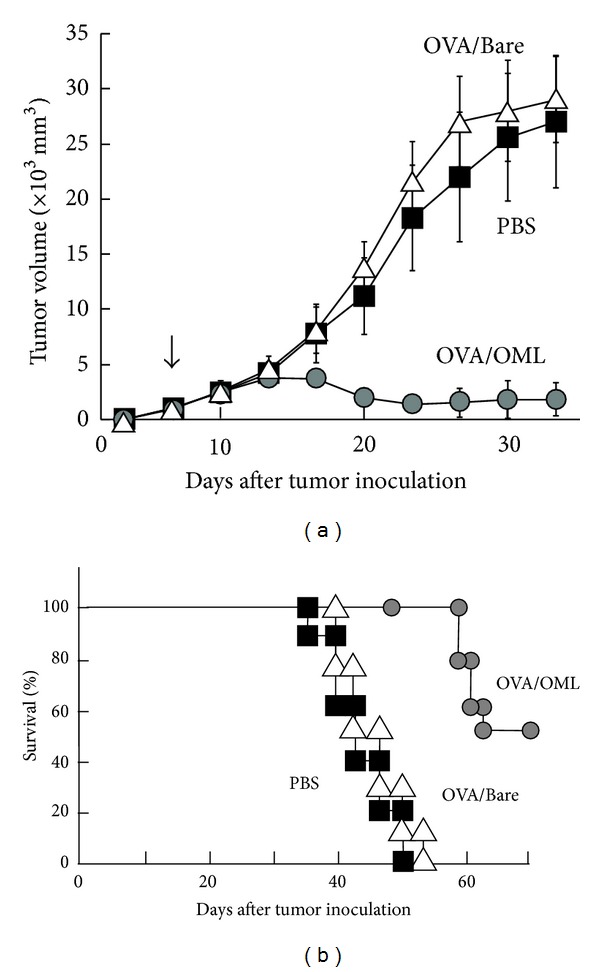
Elimination of established tumor by administration of OML-based vaccine. Mice (*n* = 25) were challenged by s.c. injection of E.G7-OVA tumor cells in the right dorsal area on day 0. On day 9, OML/OVA, Bare/OVA, or PBS was injected into the side where the tumor had grown. (a) Suppression of tumor growth. (b) Surviving mice, expressed as a percentage of the total number of mice in each group.

Since tumor-specific CTLs are the most important effector cells for antitumor immunity, an OML-based vaccine with an encased tumor antigen might be useful in antitumor therapy. Induction of antitumor immunity in an animal bearing a large tumor mass is difficult, since an immunosuppressive environment develops along with tumor growth [69]. Thus, we evaluated OML-based vaccination to assess its clinical utility in the eradication of an established tumor [24]. OML-based vaccination elicited sufficient antitumor immunity to suppress tumor growth and led to tumor rejection: a single injection of OMLs containing 1 *μ*g of antigen induced antitumor activity in model E.G7-OVA tumor-bearing mice, and about half of the mice exhibited elimination of an established E.G7-OVA tumor (Figure 4). A similar suppressive effect was not observed in mice that received empty OMLs or a mixture of soluble OVA and empty OMLs, suggesting that effective antigen delivery by antigen-carrying OMLs is important in induction of systemic immune responses.

In the majority of studies including ours, murine models were used to assess the clinical utility of the antigen delivery systems. However, assessment using human systems is essential in the consideration of clinical application. Recently, several groups have demonstrated that OMLs could generate encased-antigen-specific immune responses not only in murine system but also in human systems. Kazako and colleagues demonstrated that OMLs with a specific antigen peptide of human T cell leukemia virus-1 (HTLV-1) encased could induce HTLV-1-specific CTLs in HLA-transgenic mice and in PBMCs from HTLV-1 carriers [70]. In addition, antigenic peptide-containing OMLs can induce CTLs specific to the HLA-restricted epitopes of survivin 2B, which is expressed in many types of tumors, in PBMCs obtained from patients *in vitro* and in HLA-transgenic mice *in vivo *(personal communication). OMLs could not only deliver the protein antigens but also DNAs encoding specific antigens to APCs to induce specific immune response. Mizuuchi et al. generated a human papilloma virus gene-containing OMLs (OML-HPV) and showed that HPV-specific CTLs could be generated from PBMC of HPV-positive cervical carcinoma patients by stimulating OML-HPV [71]. These feasibility studies of OML-based vaccines using murine, bovine, and human models, as shown in Table 1, have revealed their potential for clinical use in vaccination for various diseases in which CTLs and/or Th1 cells act as effector cells.

## 8. Conclusion and Future Perspectives 

Strategies that target APCs and modulate function of APCs *in vivo* can have significant implications for vaccine design. A large number of particulate carriers are now available for antigen delivery to APCs and uptake of particulate carriers with or without surface-conjugated targeting ligands by APCs has been demonstrated in *in vitro *and* in vivo* animal studies [4, 72, 73]. The recent emergence of carriers to deliver antigens and drugs to cells by carbohydrate ligands for lectin receptors has also demonstrated the exciting potential for developing new therapeutic technology [74, 75]. For example, liposomes decorated with carbohydrate ligands for CD169 have recently been shown to be suitable for the targeted delivery of protein and lipid antigens to macrophages via CD169-mediated endocytosis [76, 77]. However, many of these carriers could not deliver the stimulatory signals to APCs and therefore require concomitant administration of additional adjuvants to activate APCs. 

In comparison to other carriers for antigen delivery to APCs, OMLs have an excellent ability in achieving concomitant delivery of antigen and stimulatory signals to professional phagocytic cells, such as DCs and macrophages, leading to APC maturation, presentation of antigens on MHC class I and class II molecules, secretion of IL-12 from APCs, and migration of APCs into lymphoid tissues from peripheral tissues, where both naïve CD4^+^ and CD8^+^ T cells are activated to generate encased-antigen-specific Th1 cells and CTLs, respectively. Therefore, OMLs themselves have the potential for clinical use in vaccination against diseases in which CTLs and/or Th1 cells act as effector cells. 

Similar to the studies of other delivery systems, the focus of the studies of OMLs was on demonstrating immune activation without focusing on the underlying mechanisms that lead to activation of APCs. For example, the molecular mechanism and signaling that IL-12 production is preferentially enhanced and IL-6 production is suppressed in OML-ingesting phagocytic cells are still unclear, although this characteristic property of OMLs might be essential for the specific induction of encased-antigen-specific cellular immune responses. An increased focus on mechanistic studies of activation of APCs with liposome-based antigen delivery systems is required for successful development. The critical factors of the liposomes for uptake and activation of APCs, for example, carbohydrate density and particle size, remain to be elucidated. We usually use OMLs consisting of DPPC, cholesterol, and Man3-DPPE at a molar ratio of 10 : 10 : 1 and with a 1 *μ*m particle size, which have the capacity to induce strong cellular immune responses and control several diseases, since nanoparticles of 500–2000 nm size were found to be more efficiently uptaken as compared to smaller size. A preliminary study suggested that OMLs with a lower carbohydrate density or a smaller particle size did not induce cellular immune responses as well as IL-12 production. Thus, optimization of the carbohydrate density on the liposome surface and of the particle size is also necessary. However, as the understanding of signaling in APCs and formulation factors that determine immune response will progress in the future, it is without doubt that clinical application of APC-targeted carbohydrate-coated liposomal delivery and activation systems will become a reality. 

## Figures and Tables

**Table 1:** Therapeutic application of OML-based vaccine in murine, bovine, and human models.

Encased materials	Responses	Animal or source	Route	References
OVA	Elimination of established E.G7-OVA tumor	C57/BL6	s.c.	[24, 53]
Crude extract of *Leishmania *	Suppression of footpad swelling by *Leishmania major* infection	Balb/c	i.p.	[22]
Recombinant NcGRA7	Protection of dams and offspring from *Neospora caninum* infection	Balb/c	s.c.	[23]
Recombinant apical membrane antigen 1	Reduction of offspring mortality from *Neospora caninum* infection	Balb/c	s.c.	[62]
Cry j 1	Prevention of IgE elevation in sera in response to Cry j 1 sensitization	Balb/c	s.c.	[25]
OVA	Induce mucosal immune responses and suppression of development of allergic diarrhea induced by oral OVA administration	Balb/c	i.n.	[67, 68]
*α*GalCer	More effective expansion of NKT cells	C57/BL6	i.p.	[60]
Recombinant NcGRA7	Suppression of serum IFN-*γ* elevation by infection of *Neospora*, suppression of *Neospora *infection in brain	Cattle	s.c.	[63]
HLA-restricted HTLV-1 Tax epitope peptide	Induction of HTLV-1-specific CTL	HLA-transgenic mouse	s.c.	[70]
	Induction of HTLV-1-specific CTL	PBMC from HTLV-1 carriers	*In vitro *	
HLA-A24-restricted surviving 2B epitope peptide	Induction of CTL specific for HLA-restricted surviving 2B epitope	PBMC from patients	*In vitro *	^ a^
	Elimination of established surviving 2B-positive tumor	HLA-transgenic mouse	s.c.	
Human papillomavirus DNA	Induction of papillomavirus-specific CTL	PBMC from carriers	*In vitro *	[71]

i.n.: intranasal.

^a^Personal communication.
